# Molecular Beam Epitaxy Growth of Quantum Wires and Quantum Dots

**DOI:** 10.3390/nano13060960

**Published:** 2023-03-07

**Authors:** Xiaohui Li, Qian Xu, Ziyang Zhang

**Affiliations:** 1 School of Physics and Information Technology, Shaanxi Normal University, Xi’an 710119, China; 2 School of Electronic and Information Engineering, Qingdao University, Qingdao 266071, China

Molecular beam epitaxy technology has a significant advantage in semiconductor technology due to its strong controllability, especially for the preparation of materials such as quantum wires and quantum dots. Chemical beam epitaxy (CBE), metal organic compound molecular beam epitaxy (MOMBE), and laser molecular beam epitaxy (L-MBE), developed by combining molecular beam epitaxy with pulsed lasers and related technology, have allowed the preparation of novel types of quantum wires. In addition, quantum dots materials have become more diversified and the development prospect of combining laser and molecular beam epitaxy technology has greatly attracted the interest of researchers. Moreover, the prepared quantum wires and quantum dots have important applications in ultrafast optics, energy, micro–nano optoelectronic devices, etc.

This Special Issue, entitled “Molecular Beam Epitaxy Growth of Quantum Wires and Quantum Dots”, covers the latest developments and achievements in the application of quantum dots and quantum wires in combination with molecular beam epitaxy and lasers, as well as the applications of quantum dots and quantum wires in ultrafast optics, micro–nano optoelectronic devices, nonlinear optics, optoelectronics, etc. These topics have been introduced in detail in eight original research articles and two reviews.

Wang et al., successfully studied the effect of dispersion on laser performance by introducing a grating pair for dispersion management, and realized different mode-locked states of different solitons from the anomalous dispersion state to the normal dispersion state. The experiment also realized a 3 dB bandwidth of the adjustable spectrum from 15.7 to 17 nm by adjusting the intracavity polarization controller (PC) [1]. Wang et al., produced a high-quality SG-SAM, which exhibits good output characteristics and can operate for a long time under high pump power compared with G-SAM. SG-SAM possesses great modulation depth and long-term stability. At the same time, by constructing a SG-SAM-based ring cavity EDF laser, a passively mode-locked pulse operation with a pulse width of 830 fs is obtained. This work shows the potential application of graphene-based passive devices in the field of optoelectronics [2]. Lin et al., described an n-ReSe_2_/p-MoTe_2_ vertical heterojunction broadband photodetector with an optical response speed of 5.6/4.2 ms. It achieves good photoelectric characteristics from the visible light to the short-wave infrared light range. Under the broad-spectrum optical response, the maximum R and D* can be obtained when the incident light wavelength is 655 nm. The values of R and D* decrease sharply with the increase in wavelength. The experimental results can facilitate the use of the TMD photodetector for long-wavelength light detection [3]. Yang et al., fabricated excellent semiconductor lasers in harmonic mode-locked fiber lasers by synthesizing titanium nitride (TiN) quantum dot (QD) materials. The experiment showed that TiN QDs are a potential material for nonlinear devices in lasers, and have great development prospect in the field of ultra-fast lasers and nonlinear optics [4]. Shu et al., synthesized Ba_2_LaTaO_6_ (BLT) and used it as a saturable absorber (SA) in fiber lasers to study its ultrafast photonics applications. Dual-wavelength solitons and dissipative solitons are obtained in the C-band. The experimental results lay a foundation for the research of double perovskites (DPs) as optical devices in the field of optics [5]. Jiang et al., optimized the growth conditions of molecular beam epitaxy (MBE), and grew multiple five-QD layers with a large point size non-uniform distribution, precisely controlling the cavity mirror loss of the ground state (GS) and excited states (ESs). A broad and high power QD-SLED with multiple GS, ES_1_, and ES_2_ emissions, with a small dip of 1.3 dB, was realized, exhibiting a high RT-CW output power of 40 mW and a wide bandwidth of over 90 nm. The experimental results give rise to novel manufacturing technology of high-performance, low-coherence light sources based on QDs [6]. Zhang et al., used plasma-assisted molecular beam epitaxy to study the self-assembly growth of blue-green-yellow-red InGaN QDs on GaN templates, and achieved InGaN QDs with light emission wavelengths ranging from 460 nm to 622 nm. A 505 nm green light-emitting diode (LED) based on InGaN/GaN MQDs was prepared [7]. Wu et al., studied the surface dynamics on patterned GaN substrate stripes. The simulation results based on the step motion model successfully explain the growth behavior on the fringe. The experimental results show the importance of controlling the surface dynamics in GaN growth, and lay a foundation for the surface dynamics in the controller [8]. Cao et al., summarized the latest application of semiconductor 2D materials in MIR optoelectronic devices, providing a suitable class of 2D materials for light-emitting devices, modulators, and photodetectors in the MIR band, such as BP, TMDC, GDY, and 2D Te nanochips. On this basis, the challenges and prospects are summarized [9]. Yao et al., reviewed the development of semiconductor quantum dot molecular beam epitaxy (MBE) growth methods, material properties, and device properties. At the same time, three types of III–V QD-based lasers for optical communication are summarized: active electrically pumped lasers, passive lasers, and semiconductor saturable absorption mirror mode-locked lasers. This review presents the challenges in the field of optical fiber communication based on III–V QD lasers and the driving force behind its development [10].

The ten selected articles in this Special Issue cover the latest research and developments of quantum dots and quantum wires combined with molecular beam epitaxy and ultra-fast laser applications. Based on the current development, these have great potential in the field of optoelectronics, such as the application of mode-locked fiber lasers, photodetectors, nonlinear optics, nanodevices, and light sources of different wavelengths. Breakthroughs are being made in these new studies, though they still face severe challenges; thus, continuous efforts in the research and exploration of this field are required.
